# Microwave-Assisted Knoevenagel-Doebner Reaction: An Efficient Method for Naturally Occurring Phenolic Acids Synthesis

**DOI:** 10.3389/fchem.2018.00426

**Published:** 2018-09-19

**Authors:** Louis M. M. Mouterde, Florent Allais

**Affiliations:** Chaire ABI, AgroParisTech, CEBB, Pomacle, France

**Keywords:** p-hydroxycinnamic acids, ferulic acid, Sinapic acid, Caffeic acid, Coumaric acid, Knoevenagel-Doebner, microwaves

## Abstract

The common chemical method to synthesize Phenolic Acids (PAs) involves a relatively considerable energy intake. In order to solve this issue, microwave-assisted Knoevenagel-Doebner condensations were developed. Nevertheless, these synthetic procedures prove difficult to reproduce. Herein, we developed and optimized—by using a combination of a Design of Experiment and a standard optimization approach—a reliable procedure that converts naturally occuring *p*-hydroxybenzaldehydes into the corresponding PAs with conversions of 86–99% and in 85–97% yields.

## Introduction

Naturally occurring phenolic acids (PAs) are key compounds involved in the metabolism of plants and necessary building blocks for the polyphenol biosynthetic pathways to lignins, coumarins, lignans, stilbenes, and many other families of phenolic compounds (Shahidi and Nazck, 2004). Among other biological roles, PAs bridge lignin and polysaccharides (hemicellulose) in plants cell wall through ester linkages (Bach et al., 1992). The most commonly accepted routes for their biosynthesis begin with the shikimate pathway that leads to phenylalanine and tyrosine (Herrmann and Weaver, 1999). Two different pathways, involving phenylalanine ammonia-lyase (PAL) and tyrosine ammonia-lyase (TAL), respectively, metabolize these PAs yielding to *p*-coumaric, caffeic, ferulic, 5-hydroxyferulic and sinapic acid (Koukol and Conn, 1961; Potts et al., 1974; Rösler et al., 1997).

The industrial interest for naturally occurring PAs is increasing due to the emergence of promising ferulic- and sinapic-acid–based functional additives [e.g., bisphenol A substitutes (Jaufurally et al., 2016; Janvier et al., 2017), antioxidants (Reano et al., 2015, 2016b)], anti-UV molecules (i.e., sinapoyl malate), (Allais et al., 2009; Dean et al., 2014; Luo et al., 2017) and renewable polymers and resins (Kreye et al., 2013; Ouimet et al., 2013; Barbara et al., 2015; Pion et al., 2015; Hollande et al., 2016; Maiorana et al., 2016; Reano et al., 2016a; Menard et al., 2017; Nguyen et al., 2017). Encouraging works have been conducted for the biosourcing of PAs. However, extracting naturally occurring PAs from raw biomass remains complicated and rather expensive because of their low concentration in the biomass and the necessity to perform costly separation/purification steps. (Dupoiron et al., 2017, 2018) Thereby, the main access to naturally occurring PAs consists in the condensation of malonic acid with phenolic aldehydes that are readily obtained from the oxidation of lignins (Araújo et al., 2010; Tarabanko and Tarabanko, 2017).

In order to develop an efficient and reproducible method for the synthesis of natural occurring PAs, microwave- assisted reactions have been preferred. This methodology has been largely described in the literature, however, when it comes to Knoevenagel-Doebner condensation involving phenolic aldehydes and malonic acid, the described processes use either domestic microwave oven (Sampath Kumar et al., 1998, 2000; Mitra et al., 1999; Karchgaudhuri et al., 2002; Perez et al., 2003; Mogilaiah and Randheer Reddy, 2004; Gupta and Wakhloo, 2007; Rodrigues-Santos and Echevarria, 2007; Mobinikhaledi et al., 2008; Dhruva Kumar and Sandhu, 2010; Goel, 2012) or combined apparatus for ultrasound-microwave reactions (Peng and Song, 2003). Although this literature reports good conversions/yields, these procedures are hardly reproducible because of the use of relatively complicated and not readily accessible instrument, or of non-scientific ovens (i.e., household microwave oven) that are difficult to rely on. For instance, applying the milder conditions reported in the literature (time: 1 min, power: 300W) (Goel, 2012) with a scientific equipment, such as the Monowave 400 from Anton Paar, in different solvents (DMF, EtOH, water), resulted in a quantitative decomposition of malonic acid into acetic acid (≥99%). Therefore, it became urgent to develop a reliable method for microwave-assisted Knoevenagel-Doebner condensation of naturally occurring PAs with malonic acid.

## Results and discussion

**Scheme 1:**
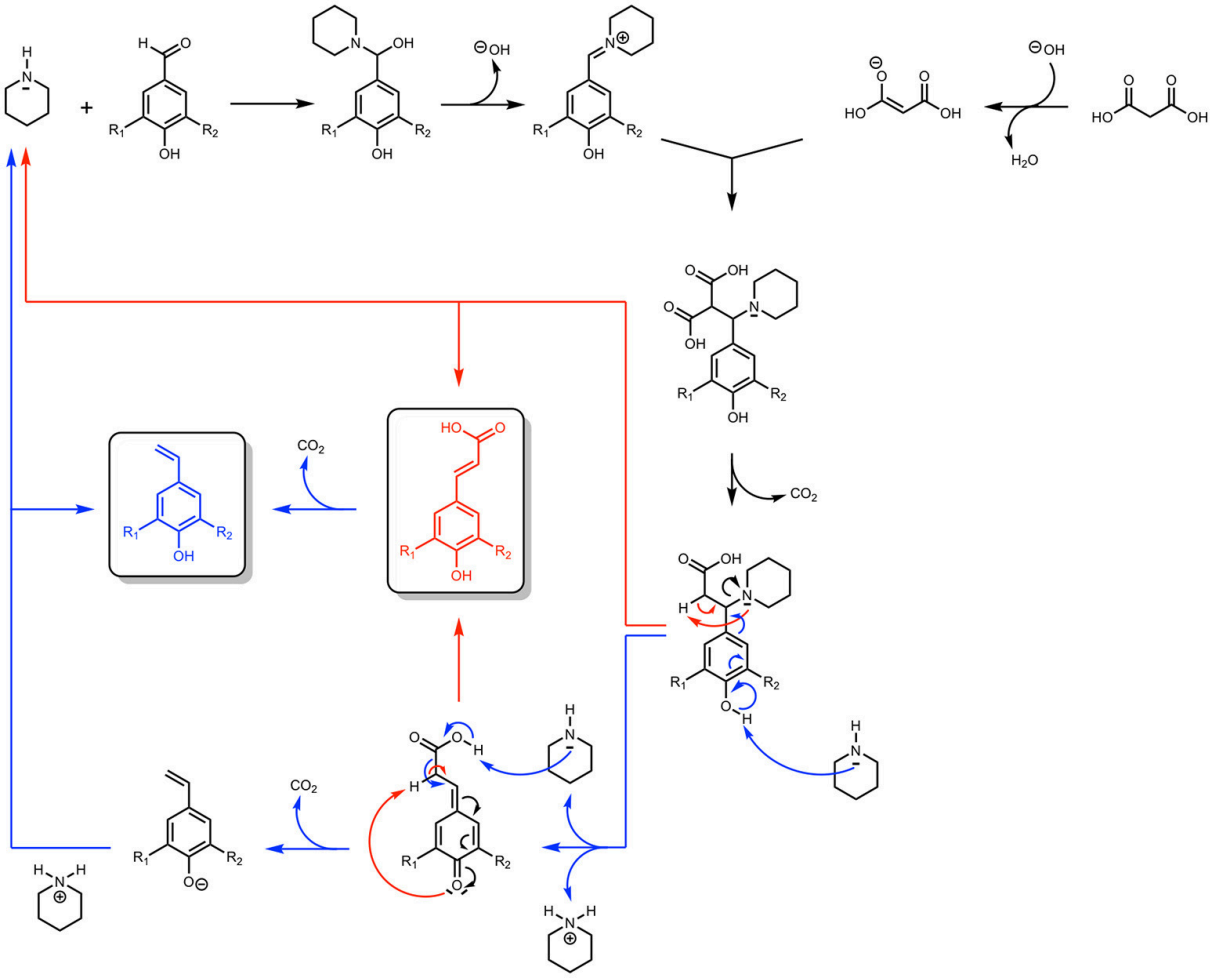
Knoevenagel-Doebner condensation of *p*-hydroxybenzaldehydes using malonic acid and piperidine (Proposed mechanism).

Performing Knoevenagel-Doebner condensation in presence of malonic acid poses another challenge, namely the decarboxylation of PAs to the corresponding vinyl phenols in either thermal and irradiated conditions (Scheme 1). (Sinha et al., 2007; Zago et al., 2015) Indeed, these works have shown that, depending on the reaction conditions, the Knoevenagel-Doebner condensation on *p*-hydroxycinnamaldehydes can result in a mixture of the corresponding PAs but also their vinylphenols. Many mechanisms have been proposed to account for this outcome and a theoretical thermodynamical study performed by Bermúdez et al. (2010) has demonstrated that the most relevant one involves piperidine as a leaving group that can promote either the formation of the PAs or that of the vinylphenols. Not only this mechanism shows that an excess of piperidine is needed to access vinylphenols (blue route in Scheme 1), but also the calculations proved that the reaction must be heated at relatively high temperatures to overcome the unfavorable thermodynamic and kinetic factors of the formation of PAs and their corresponding vinylphenols.

With regards to this potential side reaction, the use of no more than 1 equivalent of base was thus preconized, and in accordance with classical Knoevenagel-Doebner conditions (Pawar et al., 2016), the reaction was first carried out in toluene (5 mL) using vanillin—as model—in presence of 3 equivalents of malonic acid and 0.25 equivalent of piperidine. The microwave irradiations were set at 50 W until reaching 120°C, followed by a temperature hold of 20 minutes. ^1^H & ^13^C NMR analysis of the crude reaction mixture revealed that vanillin was converted into ferulic acid (60% yield). However, the decarboxylation of ferulic acid into 2-methoxy-4-vinylphenol also occurred (19% yield). In order to have a better control of the reaction, the influence of various parameters/variables (i.e., temperature, equivalent of piperidine, equivalent of malonic acid and reaction time) were investigated by first performing a design of experiment (DoE) based on Response Surface methodology (RSM), an effective statistical approach for optimizing a range of synthetic procedures and evaluating the interactions of multiple reaction parameters (Table 1) (Mongtgomery, 2008).

**Table 1:** Variables and their variation corresponding to defined levels of the DoE.

	**Levels**		
**Variables**	**−1**	**0**	**1**
Temperature (°C)	100	120	140
Time (min)	10	20	30
Malonic acid equivalents	2	3	4
Piperidine equivalents	0.125	0.1875	0.25

To find a relationship between theses variables and response surface, the following second-order polynomial equation was used:

(1) $$\begin{array}{r} \text{Y}={\text{α}}_{0}+\underset{i}{\sum }\alpha_{i\;}x_{i}+\underset{i}{\sum }\alpha_{j\;}{x_{i}}^{2}+\;\underset{ij}{\sum }\alpha_{ij\;}x_{i}x_{j} \end{array}$$

where Y represents the response (conversion), x_i_ are the variables, α_0_ is a constant and α_i_, α_j_, and α_ij_ are the linear, quadratic and interaction coefficients. Regression coefficients were determined by multiple linear regressions (MLR). The significant parameters in the model were found by analysis of their *p*-value (< 0.05).

The model validation was based on the variance (ANOVA) for each response, namely, by the analysis of R^2^, Q^2^, and *lack of fit* (LOF) test. R^2^ measures how well the regression model fits the experimental data, Q^2^ shows an estimate of the future prediction precision, and LOF assesses whether the models error is comparable to the replicate error. A D-optimal design consisting in 28 experiments, including 3 three replications at the central point to evaluate their reproducibility, was used to determine the optimum set of experimental parameters to optimize the reaction conversion (see Supporting Information for D-optimal design values).

**Scheme 2:**
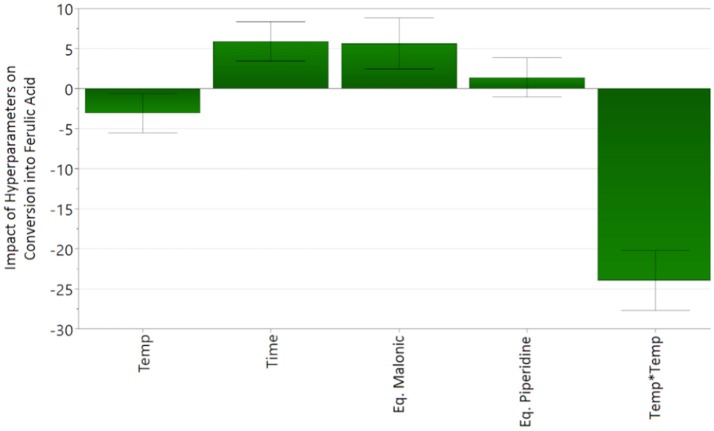
Regression coefficients of the quadratic model, Equation 1.

The experimental data of the D-Optimal design was fitted to the second-order polynomial equation (Eq. 1). Analysis of variance (ANOVA) shows a good correlation of the second-order polynomial model between the response (conversion) and the significant variables (*p* < 0.05). The *p*-value of the regression model below 0.05 shows the statistical significance of the polynomial regression. The *lack of fit* (*p* > 0.05) shows the low replicate errors of the model. Finally, the design gives a very good coefficient of determination (R^2^ = 0.952 > 0.5) and acceptable coefficient of cross-validation (*Q*^2^ = 0.868 > 0.5) that corresponds to a good fit and prediction of the model. Scheme 2 shows the coefficients (α_0_ as constant, α_i_, α_j_ and α_ij_ of the Eq. 1) of the model for the response. A positive value means a positive influence on the conversion while a negative value means a negative influence.

**Scheme 3:**
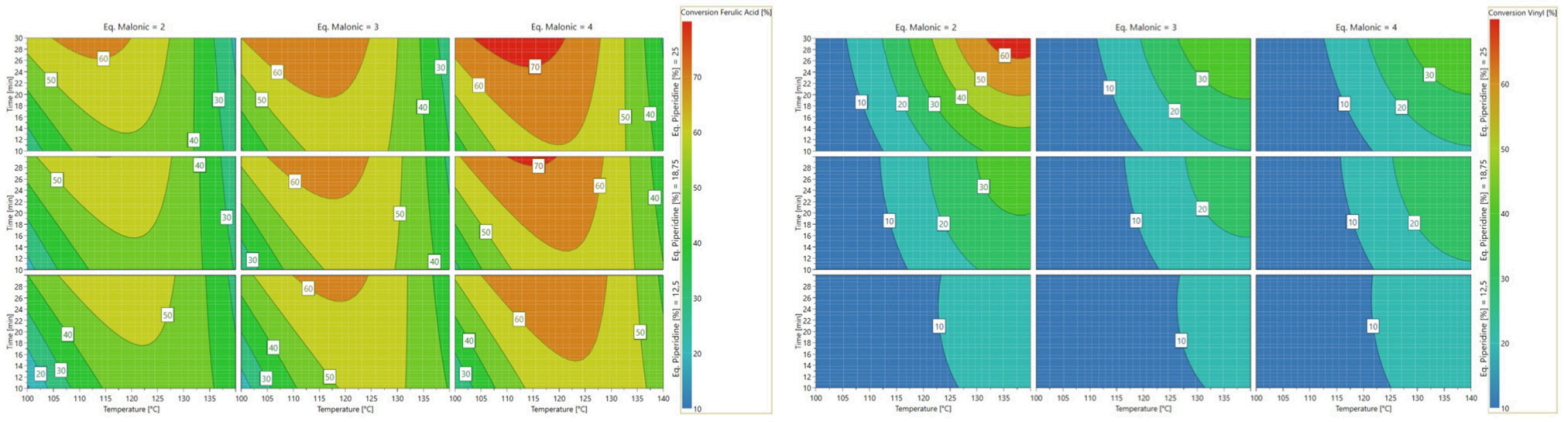
**(Left)** Contour plots of vanillin conversion into ferulic acid. **(Right)** Contour plots of ferulic acid conversion into 2-methoxy-4-vinylphenol.

Thereby, results in Scheme 2 show that the reaction time and the number of equivalents of malonic acid have a positive influence on the conversion of vanillin into ferulic acid whereas temperature has a negative one, with optimum values on the intervals of study according to their respective quadratic terms. Moreover, temperature interacts negatively with both time and number of equivalent of piperidine. This behavior is in accordance with the know fact (Sinha et al., 2007; Zago et al., 2015) that the decarboxylation of phenolic acids occurs at high temperature in presence of an excess of bases (Scheme 2, Temp^*^Eq. Piperidine and Scheme 3). Taken alone, the influence of the number of equivalent of piperidine is not significant on the ferulic acid conversion rate (*p*-value > 0.05). Finally, the equation of the model (Eq. 2) is as follows:

(2) $$\begin{array}{l} \text{Conversion into ferulic acid}(\%)=\text{58}.\text{22−3}.\text{09}\times \text{Temp+5}.\text{89}\\ \;\times \text{Time+5}.\text{63}\times \text{Eq}.\text{malonic acid}\\ \text{ −23}.\text{96 }\times {\text{Temp}}^{\text{2}}\text{−9}.\text{59}\times ({\text{Temp}}^{*}\text{Time})\\ \text{ −5}.\text{80}\times ({\text{Temp}}^{*}\text{Eq}.\text{piperidine}) \end{array}$$

The analysis of the 4D response contour allowed the visualization of the influence of each parameter (Scheme 3). In the interval considered, thanks to Eq. 2, the optimal temperature and reaction time are evaluated at 120°C and 17 min, respectively, and the optimal number of equivalents of malonic acid and piperidine are 4 and 0.25, respectively. Applying these conditions allowed the production of ferulic acid in 67% yield while minimizing the formation of 2-methoxy-4-vinylphenol (4%), demonstrating that, under optimized conditions, decarboxylation is limited.

As the optimal conversion obtained using the DoE is relatively low, other factors are certainly involved. Looking deeper into the experimental results of the DoE, we found out that the reaction did not go further because of the degradation of malonic acid into acetic acid (specific signals of acetic acid were identified in the ^1^H spectrum of the crude reaction). One solution to this problem would be to perform the reaction with more equivalents of malonic acid nevertheless this would go against reaction mass efficiency. Thus, other parameters were varied in order to improve this conversion rate.

In a first place, doubling the concentration of the reaction medium and the number of equivalent of piperidine resulted in a relatively small increase of the conversion rate (70 vs. 67%, Table 2–Entry 2 vs. Entry 1) but also favored the formation of vinyl-phenol (12 vs. 4%). Lowering the reaction temperature (90°C) and applying a longer reaction time (30 min) solved this issue and provided the same conversion rate of ferulic acid (72%, Table 2–Entry 3) but with almost no formation of the corresponding 2-methoxy-4-vinylphenol (2%). In accordance with the results from the DoE and the work of Bermúdez et al. (2010), this set of experiments confirms that the decarboxylation of ferulic acid into 2-methoxy-4-vinylphenol is promoted when the reaction is performed at high temperature (120°C) and with an excess of piperidine^1^.

**Table 2:** Optimization of the microwave-assisted Knoevenagel-Doebner condensation on vanillin at 50 W.

**Entry**	**Base**	**Eq. of base**	**Solvent**	**Concentration (M)**	**Temperature (^°^C)**	**Time (min)**	**% Ferulic acid**	**% Vinyl Phenol**
1	Piperidine	0.25	Toluene	0.8	120	17	67	4
2	Piperidine	0.5	Toluene	1.6	120	17	70	12
3	Piperidine	0.5	Toluene	1.6	90	30	72	2
4	NEt_3_	0.5	Toluene	1.6	90	30	47	5
5	DBU	0.5	Toluene	1.6	90	30	57	7
6	K_2_CO_3_	0.5	Toluene	1.6	90	30	21	1
7	Piperidine	0.5	DMF	1.6	90	30	92	4
8	Piperidine	0.5	Cyrene®	1.6	90	30	63	0
9	Piperidine	0.125	DMF	1.6	90	30	42	0
10	Piperidine	0.25	DMF	1.6	90	30	81	1
11	Piperidine	0.625	DMF	1.6	90	30	81	17

Different bases, NEt_3_, DBU, and K_2_CO_3_, were then tested. As they led to lower ferulic acid yields (47, 57, and 21%, respectively; Table 2, Entries 4–6) and similar or higher vinylphenol yields (5, 7, and 1%; Table 2, Entries 4–6), we decided to keep piperidine as the base and to focus on the solvent. Another solvent with better solubilizing ability than toluene was considered and the reaction was thus carried out in DMF at 90°C. This resulted in the increase of the conversion rate (from 72% to Table 2, Entry 3 to 92%–Table 2, Entry 7) without significantly impacting the vinylphenol yields (from 2%–Table 2, Entry 3 to 4%–Table 2, Entry 7). The use of a greener solvent has also been explored. Indeed, recent work showed that Cyrene®, a bio-sourced solvent obtained from the hydrogenation of cellulose-derived levoglucosenone (Shafizadeh et al., 1978), can be a good substituent for toxic dipolar aprotic solvent such as NMP, DMF, or sulpholane (Sherwood et al., 2014). Unfortunately, replacing DMF by Cyrene® did not lead to comparable conversion rates (63%, Table 2–Entry 8). As previously discussed, the number of equivalent of piperidine has a beneficial effect on the conversion of vanillin into ferulic acid until a certain level at which it started to favor its conversion into the corresponding vinyl-phenol by promoting the decarboxylation. Following this observation, the reaction from Entry 7 was performed with number of equivalents of piperidine from 0.125 to 0.625 (Table 2, Entries 9–11) and data confirmed that with 0.625 equivalent of piperidine, ferulic acid was readily converted into 2-methoxy-4-vinylphenol up to 17% yield. To limit this undesired side-reaction, the optimal quantity of piperidine was then kept at 0.5 equivalent.

In summary, the optimal reaction conditions are: DMF (1.6 M), 0.5 eq. piperidine, 3 eq. malonic acid, 50 Watts to 90°C and a 30 mins hold a this temperature (Entry 7, Table 2). Applying these conditions to the other naturally occurring substituted *p*-hydroxybenzaldehydes (i.e., 4-hydroxybenzaldehyde, 3,4-dihydroxy-benzaldehyde, vanillin, dihydroxyvanillin, syringaldehyde) provided the corresponding PAs in very good yields while limiting the formation of the corresponding vinyl phenols through decarboxylation (Table 3).

**Table 3:** Conversion rates and yields of natural occurring phenolic acids.

**Substrates**	**Phenolic acids**	**% Conversion^a^**	**% Yield^b^**
4-Hydroxybenzaldehyde	*p*-Coumaric acid	96	92
3,4-Dihydroxybenzaldehyde	Caffeic acid	86	85
Vanillin	Ferulic acid	92	89
5-Hydroxyvanillin	5-Hydroxyferulic acid	90	87
Syringaldehyde	Sinapic acid	93	90

a *Conversion were determined by ^1^H NMR of the crude reaction mixture*.

b *Yields were calculated from isolated product after purification*.

## Conclusion

An efficient and reliable microwave-assisted Knoevenagel-Doebner condensation of naturally occurring *p*-hydroxy-benzaldehydes with malonic acid has been developed and optimized through the combination of a design of experiment and a standard optimization approach, providing the corresponding naturally occurring phenolic acids with conversion rates from 86 to 96% and yields from 85 to 92%. This optimized microwave-assisted synthetic route offers a reliable and reproducible Knoevenagel-Doebner condensation while limiting the decarboxylation of the resulting phenolic acids.

## Experimental section

### General

Microwave reactions were carried out into a Monowave 400 Anton Paar® system. Evaporations were conducted under reduced pressure at 65°C. ^1^H NMR spectra of samples in the indicated solvent were recorded at 300 MHz at 20°C [^1^H NMR: (CD_3_)_2_CO residual signal at δ = 2.05 ppm]. ^13^C NMR spectra of samples in the indicated solvent were recorded at 75 MHz at 20°C [^13^C NMR: (CD_3_)_2_CO residual signal at δ = 206.26 and 29.84 ppm]. The atomic labeling used in the assignment of NMR signals is presented in the Supporting Information. All reported yields are uncorrected and refer to purified products. All reagents were purchased from Sigma-Aldrich or TCI and used without further purification.

### Optimized method for the synthesis of PAs

The corresponding phenolic aldehydes (8 mmol) and malonic acid (24 mmol, 2.5 g) were mixed together into DMF (5 mL) until complete dissolution. Piperidine (4 mmol, 400 μL) was then added to the reaction mixture, the tube sealed and placed into a Monowave 400. Constant power (50 W) was applied until reaching a temperature of 90°C which was then maintained for 30 additional minutes. The reaction mixture was then evaporated under vacuum and the desired product precipitated by addition of 50 mL of cold diluted aq. NH_4_Cl solution.

### *p-*Coumaric acid

^1^H NMR (300 MHz, (CD_3_)_2_CO): δ = 7.61 (d, *J* = 15.96 Hz, 1H, H-3), 7.57 (s, 1H, H-5 or H-9), 7.54 (s, 1H, H-5 or H-9), 6.91 (s, 1H, H-6 or H-8), 6.88 (s, 1H, H-6 or H-8), 6.34 (d, *J* = 15.93 Hz, 1H, H-2). ^13^C NMR [75 MHz, (CD_3_)_2_CO]: δ = 168.3 (s, C-1), 160.5 (s, C-7), 145.6 (d, C-3), 130.9 (d, C-5 and C-9), 127.0 (s, C-4), 116.7 (d, C-2), 115.7 (d, C-6 and C-8). All analytical data were in agreement with the literature values.

### Caffeic acid

^1^H NMR [300 MHz, (CD_3_)_2_CO]: δ = 7.55 (d, *J* = 15.9 Hz, 1H, H-3), 7.16 (d, *J* = 2.04 Hz, 1H, H-9), 7.04 (dd, *J* = 2.01 and 8.22 Hz, 1H, H-6), 6.86 (d, *J* = 8.16 Hz, 1H, H-5), 6.27 (d, *J* = 15.9 Hz, 1H, H-2). ^13^C NMR [75 MHz, (CD_3_)_2_CO]: δ = 168.2 (s, C-1), 148.6 (s, C-7), 146.2 (s, C-8), 145.9 (d, C-3), 127.5 (s, C-4), 122.4 (d, C-5), 116.2 (d, C-6), 115.6 (d, C-2), 115.1 (d, C-9). All analytical data were in agreement with the literature values.

### Ferulic acid

^1^H NMR [300 MHz, (CD_3_)_2_CO]: δ = 7.61 (d, *J* = 15.9 Hz, 1H, H-3), 7.34 (d, *J* = 1.89 Hz, 1H, H-9), 7.15 (dd, *J* = 1.86 and 8.22 Hz, 1H, H-6), 6.87 (d, *J* = 8.16 Hz, 1H, H-5), 6.39 (d, *J* = 15.93 Hz, 1H, H-2), 3.92 (s, 3H, H-11). ^13^C NMR [75 MHz, (CD_3_)_2_CO]: δ = 168.3 (s, C-1), 149.9 (s, C-8), 148.6 (s, C-7), 145.9 (d, C-3), 127.4 (s, C-4), 123.8 (d, C-5), 116.0 (d, C-2), 115.8 (d, C-6), 111.2 (d, C-9), 56.2 (q, C-11). All analytical data were in agreement with the literature values.

### 3,4-dihydroxy-5-methoxycinnamic acid

^1^H NMR [300 MHz, (CD_3_)_2_CO]: δ = 7.54 (d, *J* = 15.87 Hz, 1H, H-3), 6.90 (d, *J* = 1.59 Hz, 1H, H-5), 6.83 (d, *J* = 1.65 Hz, 1H, H-9), 6.34 (d, *J* = 15.87 Hz, 1H, H-2), 3.88 (s, 3H, H-11). ^13^C NMR [75 MHz, (CD_3_)_2_CO]: δ = 168.2 (s, C-1), 149.0 (q, C-8), 146.2 (d, C-3), 137.2 (q, C-6), 126.5 (q, C-7), 116.0 (q, C-4), 110.3 (d, C-2), 104.2 (d, C-5 and C-9), 56.4 (q, C-11). All analytical data were in agreement with the literature values.

### Sinapic acid

^1^H NMR (300 MHz, (CD_3_)_2_CO): δ = ^1^H NMR (300 MHz, (CD_3_)_2_CO): δ = 7.59 (d, *J* = 15.87 Hz, 1H, H-3), 7.02 (s, 2H, H-5 and H-9), 6.41 (d, *J* = 15.87 Hz, 1H, H-2), 3.89 (s, 6H, H-11 and H-12). ^13^C NMR [75 MHz, (CD_3_)_2_CO]: δ = 168.3 (s, C-1), 148.9 (s, C-8 and C-6), 146.3 (d, C-3), 139.3 (s, C-7), 126.1 (s, C-4), 116.1 (d, C-2), 106.7 (d, C-5 and C-9), 56.6 (q, C-11 and C-12). All analytical data were in agreement with the literature values.

## Author contributions

LM and FA: co-designed the experiments, co-wrote the manuscript. LM: performed the experiments, analyzed data.

### Conflict of interest statement

The authors declare that the research was conducted in the absence of any commercial or financial relationships that could be construed as a potential conflict of interest. The handling editor is currently co-organizing a Research Topic with one of the authors, FA, and confirms the absence of any other collaboration.

## Footnotes

^1^Performing the reaction directly on ferulic acid resulted in the production of the corresponding *vinylphenol*.
