# Corrigendum: Microwave-Assisted Knoevenagel-Doebner Reaction: An Efficient Method for Naturally Occurring Phenolic Acids Synthesis

**DOI:** 10.3389/fchem.2018.00568

**Published:** 2018-11-20

**Authors:** Louis M. M. Mouterde, Florent Allais

**Affiliations:** Chaire ABI, AgroParisTech, CEBB, Pomacle, France

**Keywords:** p-hydroxycinnamic acids, ferulic acid, Sinapic acid, Caffeic acid, Coumaric acid, Knoevenagel-Doebner, microwaves

In the original article, there was a mistake in Table 2. *Optimization of the microwave-assisted Knoevenagel-Doebner condensation on vanillin at 50 W*. as published. The values in the Eq. of base column for lines 2 to 8 are not accurate. The corrected Table 2*. Optimization of the microwave-assisted Knoevenagel-Doebner condensation on vanillin at 50 W*. appears below. The authors apologize for this error and state that this does not change the scientific conclusions of the article in any way. The original article has been updated.

**Table 2:** Optimization of the microwave-assisted Knoevenagel-Doebner condensation on vanillin at 50 W.

**Entry**	**Base**	**Eq. of base**	**Solvent**	**Concentration (M)**	**Temperature (^°^C)**	**Time (min)**	**% Ferulic acid**	**% Vinyl Phenol**
1	Piperidine	0.25	Toluene	0.8	120	17	67	4
2	Piperidine	0.5	Toluene	1.6	120	17	70	12
3	Piperidine	0.5	Toluene	1.6	90	30	72	2
4	NEt_3_	0.5	Toluene	1.6	90	30	47	5
5	DBU	0.5	Toluene	1.6	90	30	57	7
6	K_2_CO_3_	0.5	Toluene	1.6	90	30	21	1
7	Piperidine	0.5	DMF	1.6	90	30	92	4
8	Piperidine	0.5	Cyrene®	1.6	90	30	63	0
9	Piperidine	0.125	DMF	1.6	90	30	42	0
10	Piperidine	0.25	DMF	1.6	90	30	81	1
11	Piperidine	0.625	DMF	1.6	90	30	81	17

