# Supplementary material for: Microwave-Assisted Knoevenagel-Doebner Reaction: An Efficient Method for Naturally Occurring Phenolic Acids Synthesis
Source: Front Chem. 2018 Sep 19;6:426. doi: 10.3389/fchem.2018.00426 (PMC6156279; doi:10.3389/fchem.2018.00426)

SUPPORTING INFORMATION

Microwave-Assisted Knoevenagel-Doebner Reaction: An Efficient Method for Phenolic Acids Synthesis

Louis M. M. Mouterde and Florent Allais*

AgroParisTech, Chaire Agro-Biotechnologie Industrielles (ABI), CEBB, 3 rue des Rouges Terres, 51110 Pomacle, France.

Phone & Fax +33 3 52 62 04 62, E-mail florent.allais@agroparistech.fr

*Author to whom correspondence should be addressed

Contents

D-optimal design for optimization of the conversion rate (experimental values calculated accordingly the production of ferulic acid, analyzed by ^{1}H NMR) S-3

^{1}H NMR spectrum of the crude mixture of 4-Hydroxybenzaldehyde, Coumaric acid and 4-Vinylphenol S-4

^{1}H spectrum of pure Coumaric acid S-5

^{13}C NMR spectrum of pure Coumaric acid S-6

^{1}H NMR spectrum of the crude mixture of 3,4-dihydroxybenzaldehyde, Caffeic acid and 4-Vinylbenzene-1,2-diol S-7

^{1}H NMR spectrum of Caffeic acid S-8

^{13}C NMR spectrum of Caffeic acid S-9

^{1}H NMR spectrum of the crude mixture of Vanillin, Ferulic acid and 2-Methoxy-4-vinylphenol S-10

^{1}H NMR spectrum of Ferulic acid S-11

^{13}C NMR spectrum of Ferulic acid S-12

^{1}H NMR spectrum of the crude mixture of 5-hydroxyvanillin, 3,4-Dihydroxy-5-methoxycinnamic acid and 3-Methoxy-5-vinylbenzene-1,2-diol S-13

^{1}H NMR spectrum of 3,4-Dihydroxy-5-methoxycinnamic acid S-14

^{13}C NMR spectrum of 3,4-Dihydroxy-5-methoxycinnamic acid S-15

^{1}H NMR spectrum of the crude mixture of Syringaldehyde, Sinapic acid and 2,6-dimethoxy-4-vinylphenol S-16

^{1}H NMR spectrum of Sinapic acid S-17

^{13}C NMR spectrum of Sinapic acid S-18

D-optimal design for optimization of the conversion rate (experimental values calculated accordingly the production of ferulic acid, analyzed by ^{1}H NMR)

Experimental number	Factor				Conversion Acid (%)	Conversion Vinyl (%)
	Temperature (° C)	Time (min)	Eq. piperidine	Eq. malonic		
1	100	10	0.125	2	31	0
2	140	10	0.125	2	33	13
3	100	30	0.125	2	39	1
4	140	10	0.25	2	25	33
5	100	30	0.25	2	54	5
6	140	30	0.25	2	0	50
7	100	10	0.125	4	22	0
8	100	30	0.125	4	50	0
9	140	30	0.125	4	41	19
10	100	10	0.25	4	33	0
11	140	10	0.25	4	40	21
12	140	30	0.25	4	26	36
13	100	20	0.1875	3	40	1
14	140	20	0.1875	3	27	27
15	120	10	0.1875	3	45	5
16	120	30	0.1875	3	62	19
17	120	20	0.125	3	54	5
18	120	20	0.25	3	60	19
19	120	20	0.1875	2	56	20
20	120	20	0.1875	4	62	9
21	120	20	0.1875	3	62	12
22	120	20	0.1875	3	63	13
23	120	20	0.1875	3	60	14
24	110	10	0.125	2	53	6
25	110	15	0.125	3	68	18
26	100	20	0.125	3	61	5
27	100	60	0.125	3	74	17
28	110	10	0.25	3	54	6

^{1}H NMR spectrum of the crude mixture of 4-Hydroxybenzaldehyde. Coumaric acid and 4-Vinylphenol

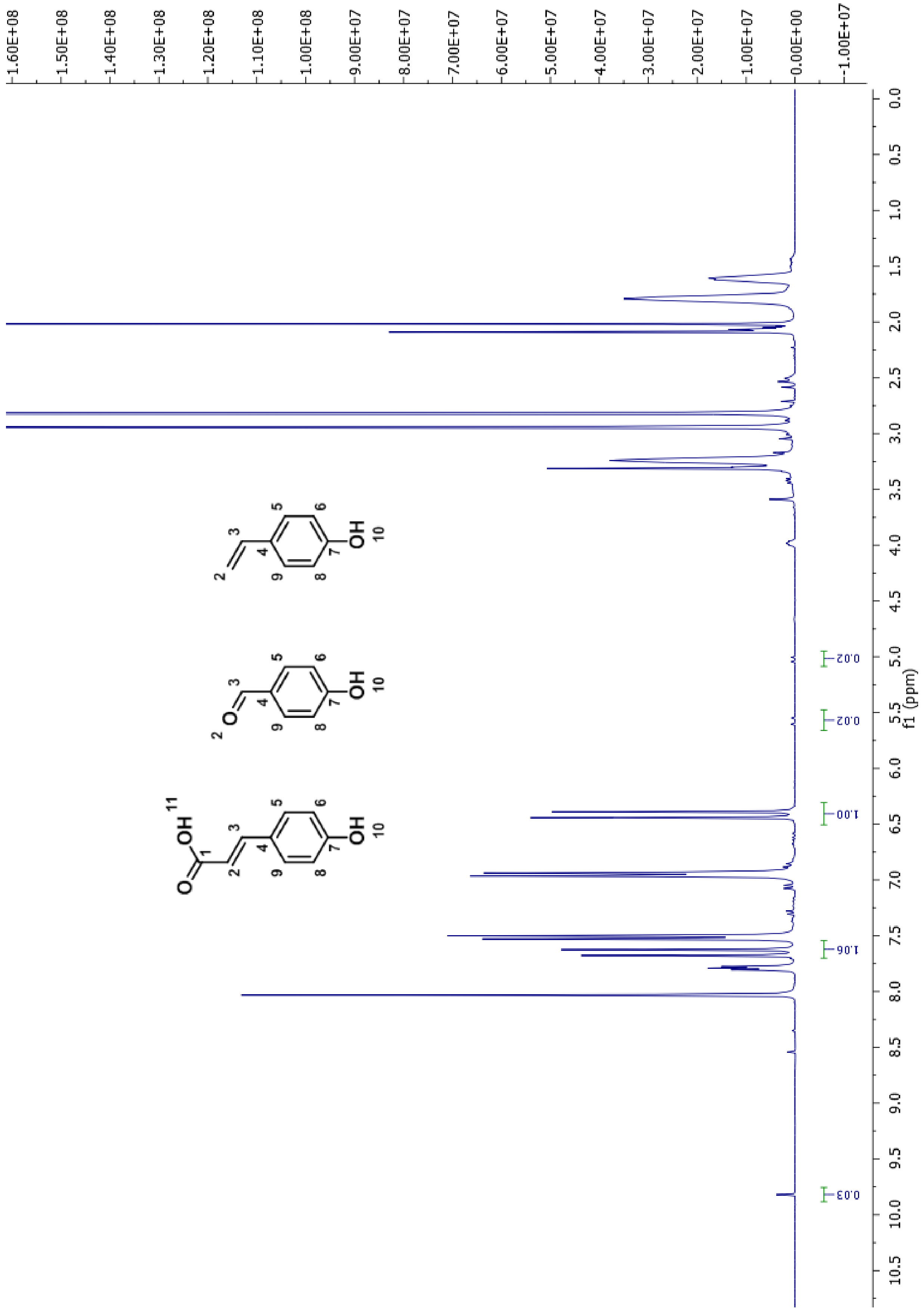

^{1}H spectrum of pure Coumaric acid

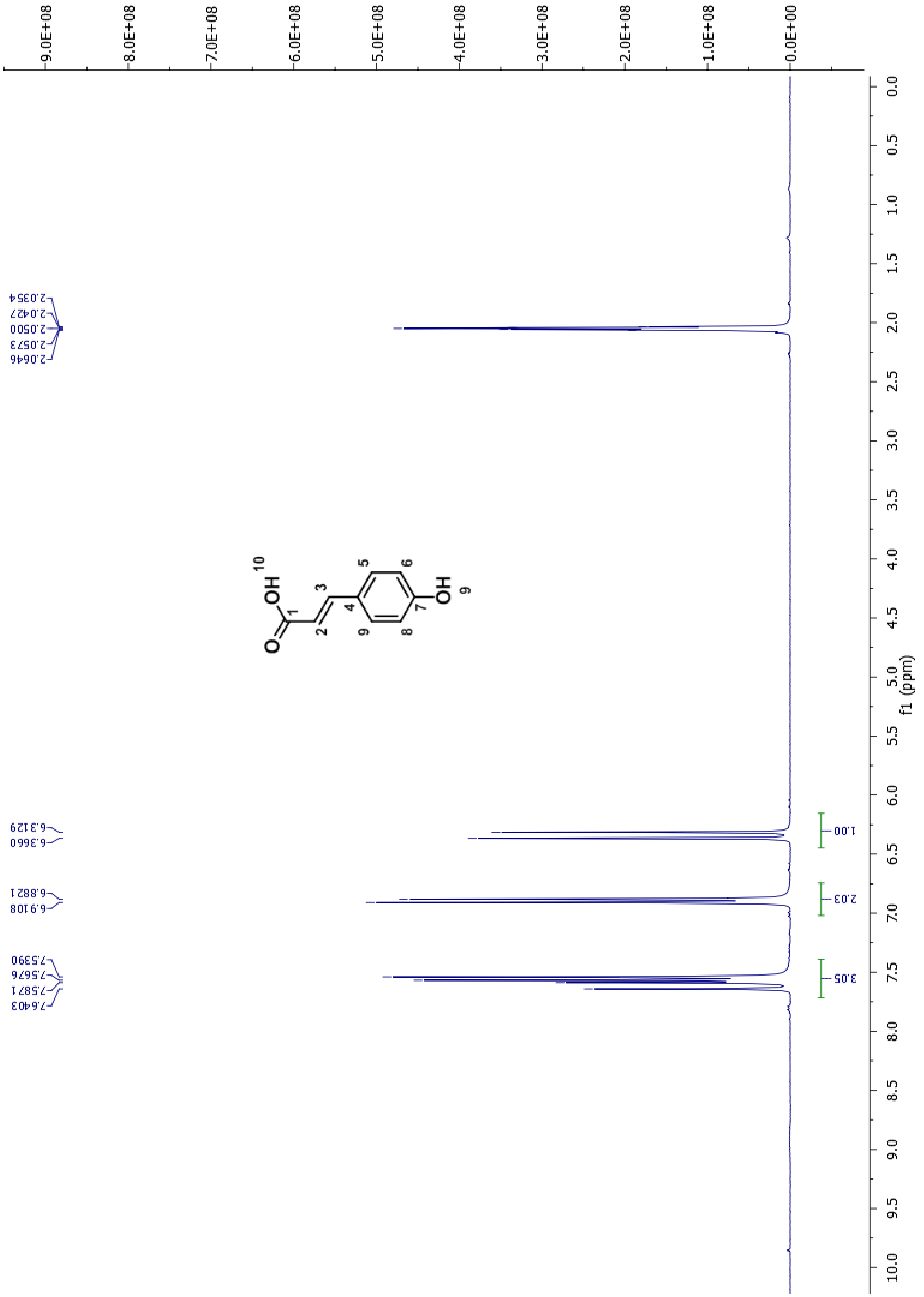

<u>^{13}C NMR spectrum of pure Coumaric acid</u>

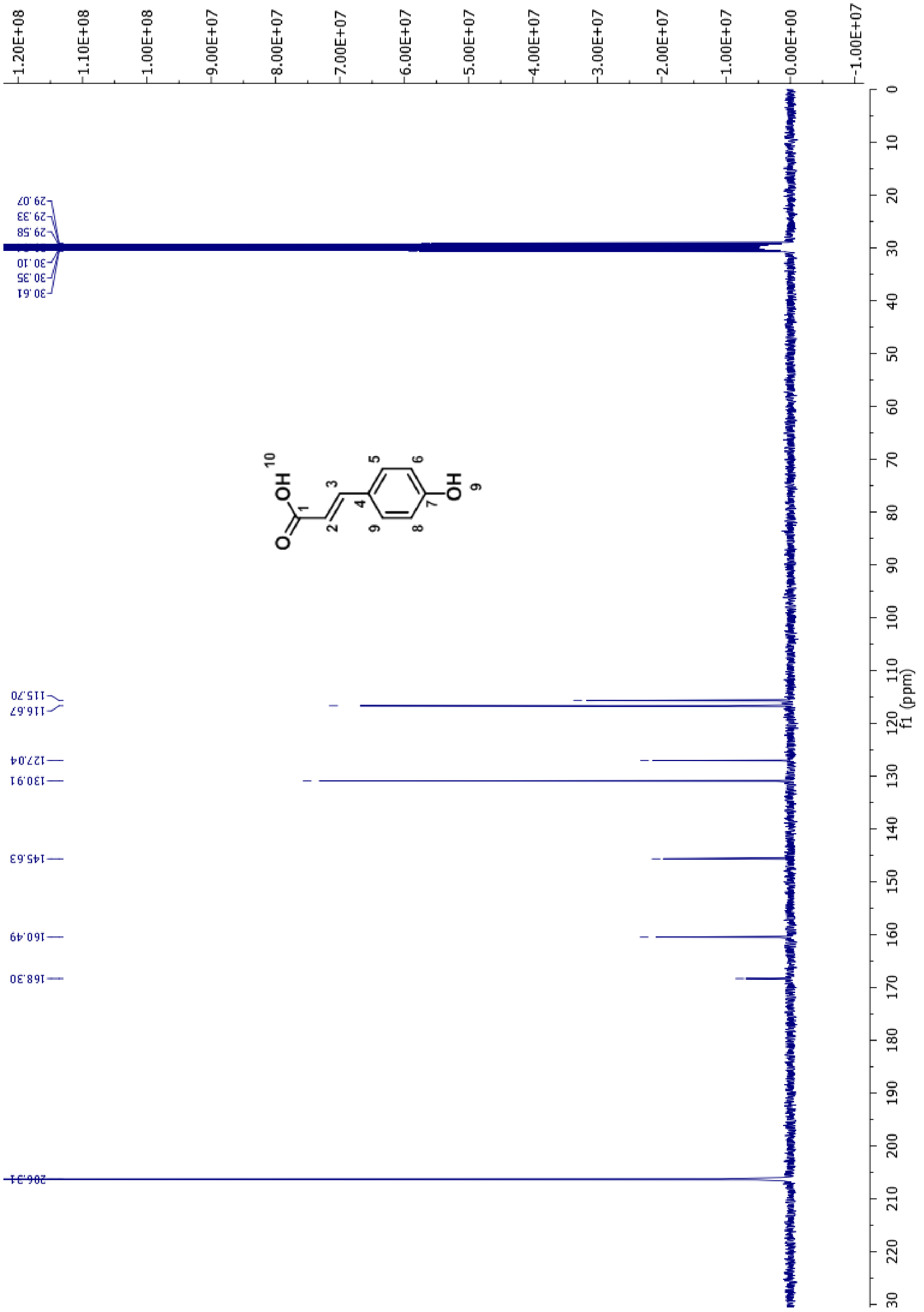

^{1}H NMR spectrum of the crude mixture of 3.4-dihydroxybenzaldehyde. Caffeic acid and 4-Vinylbenzene-1.2-diol

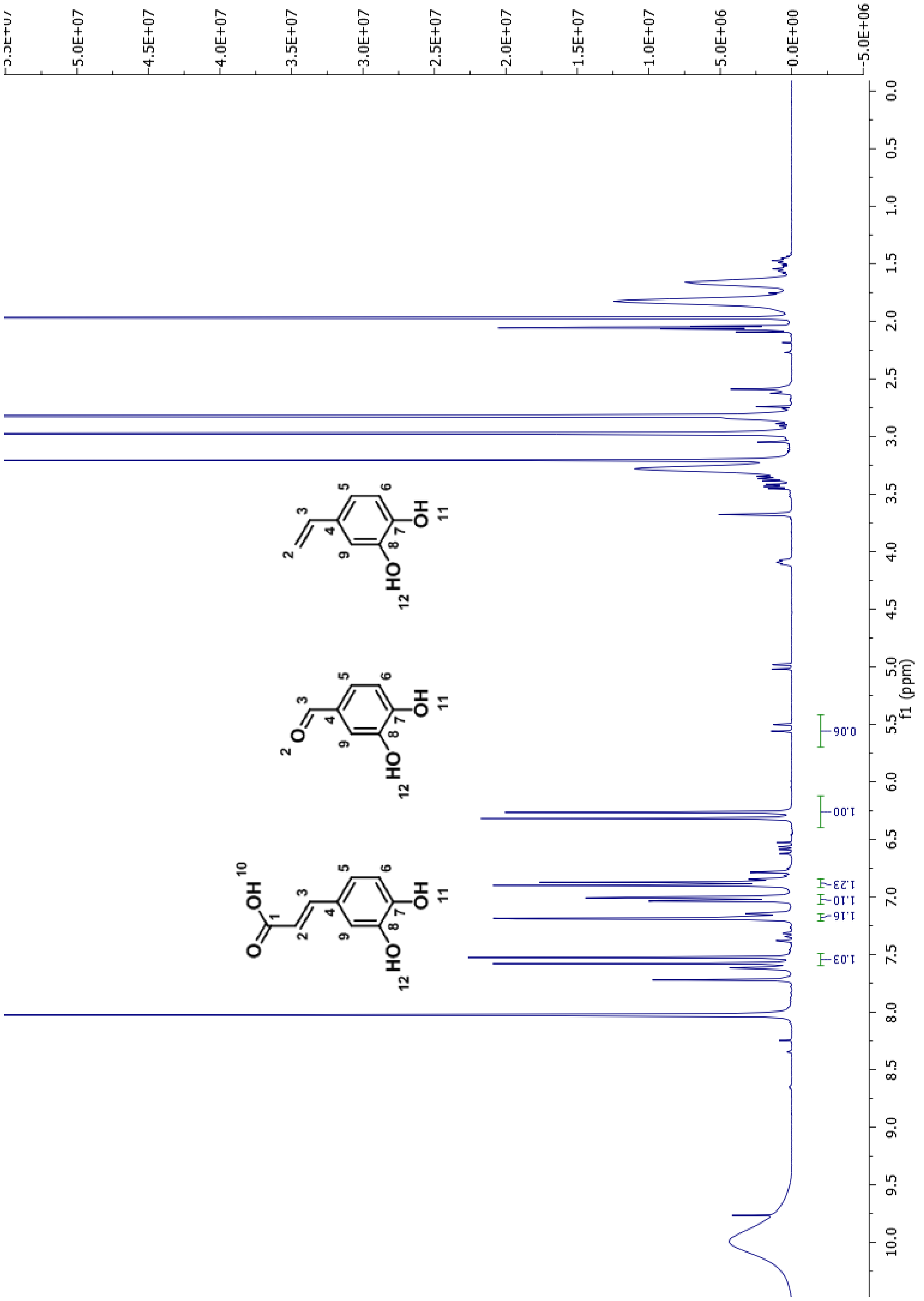

^{1}H NMR spectrum of Caffeic acid

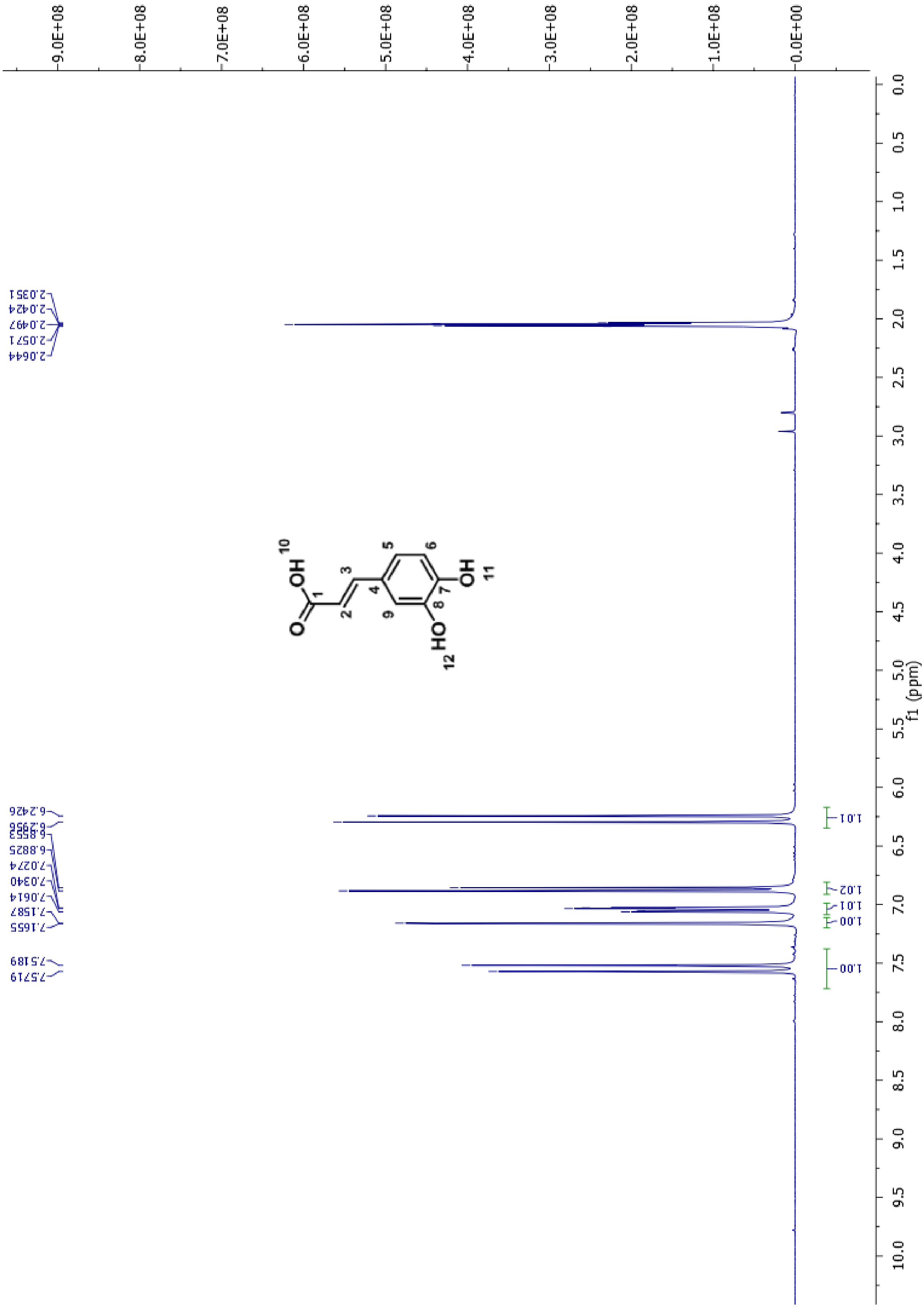

^{13}C NMR spectrum of Caffeic acid

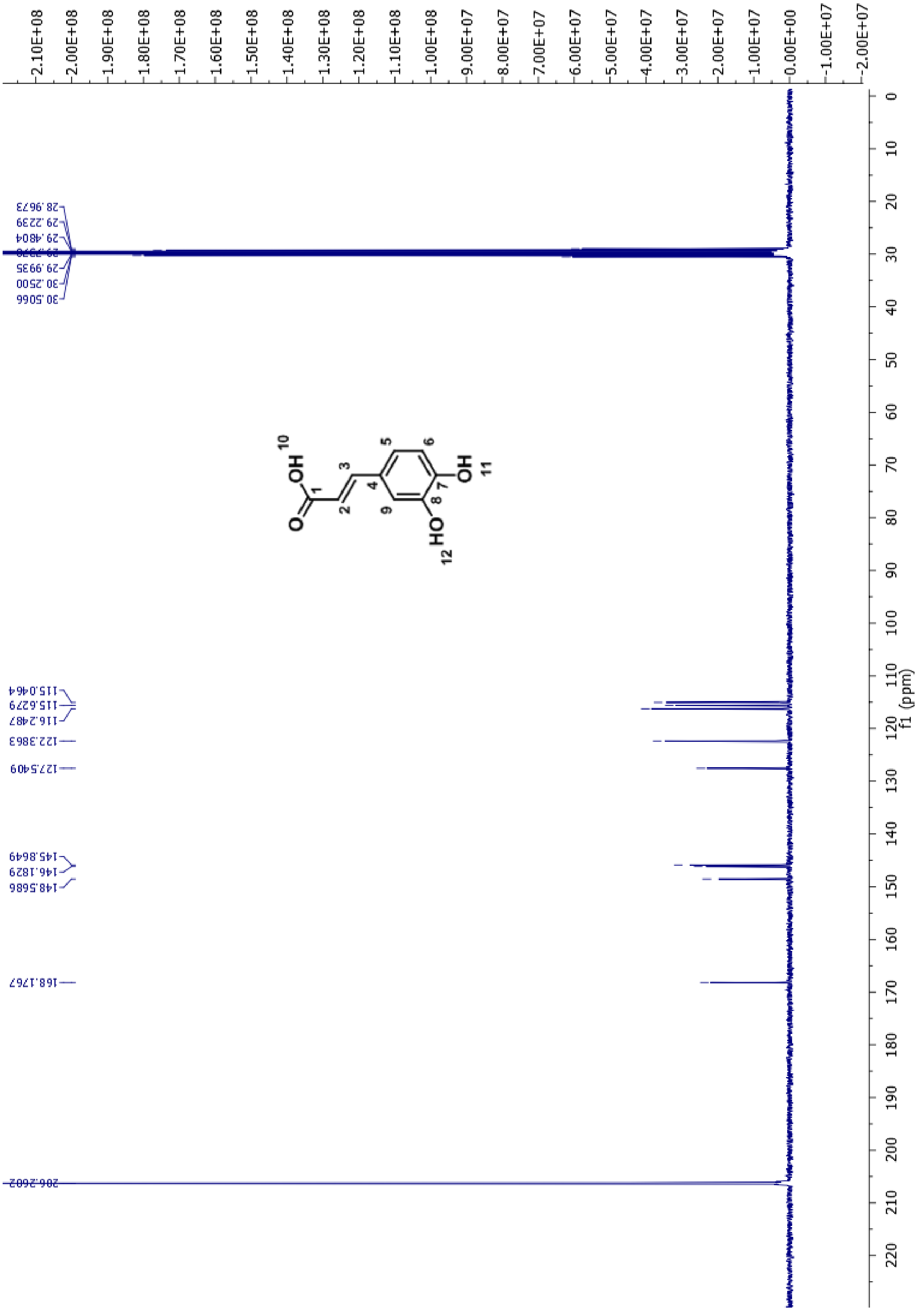

^{1}H NMR spectrum of the crude mixture of Vanillin. Ferulic acid and 2-Methoxy-4-vinylphenol

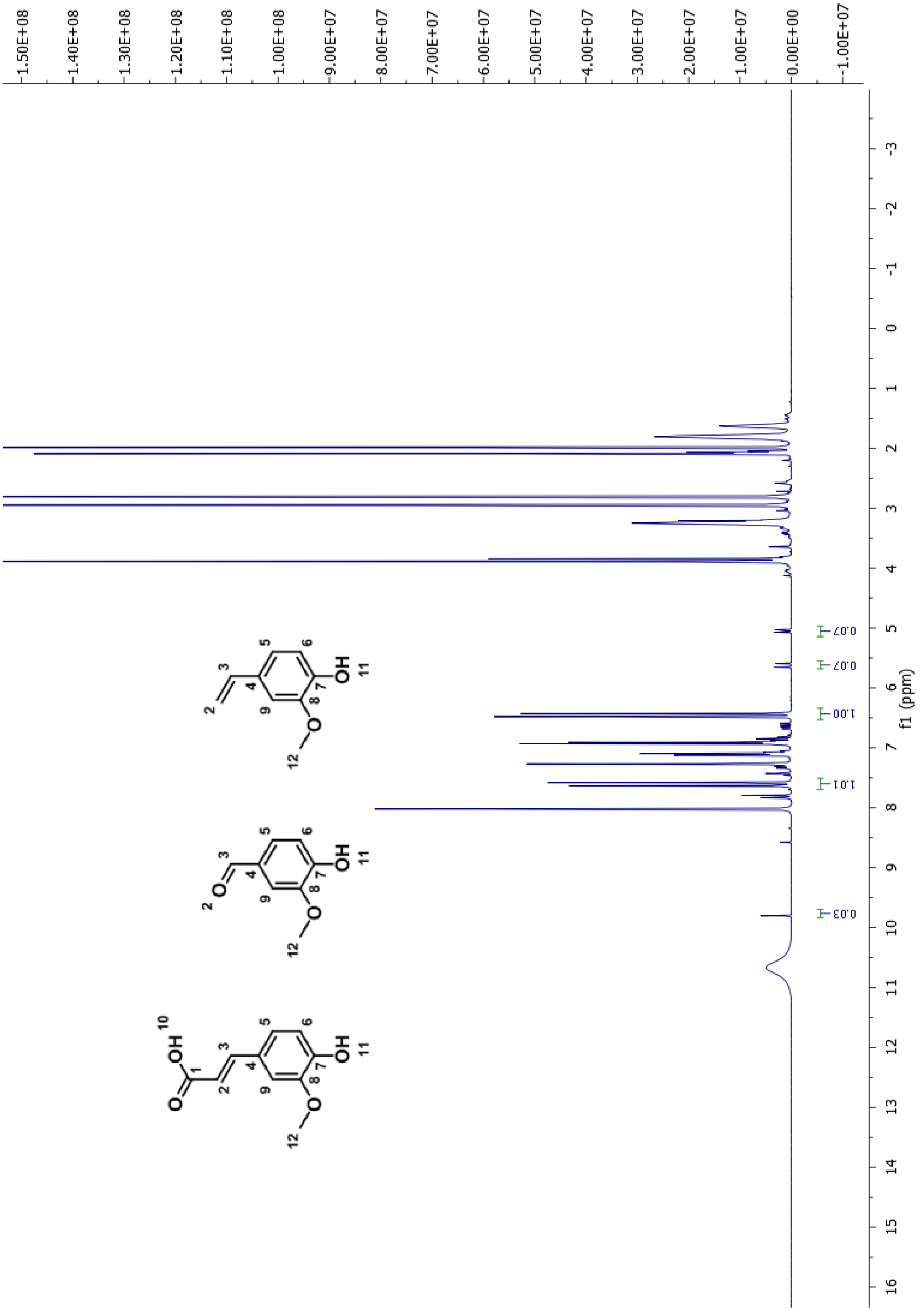

^{1}H NMR spectrum of Ferulic acid

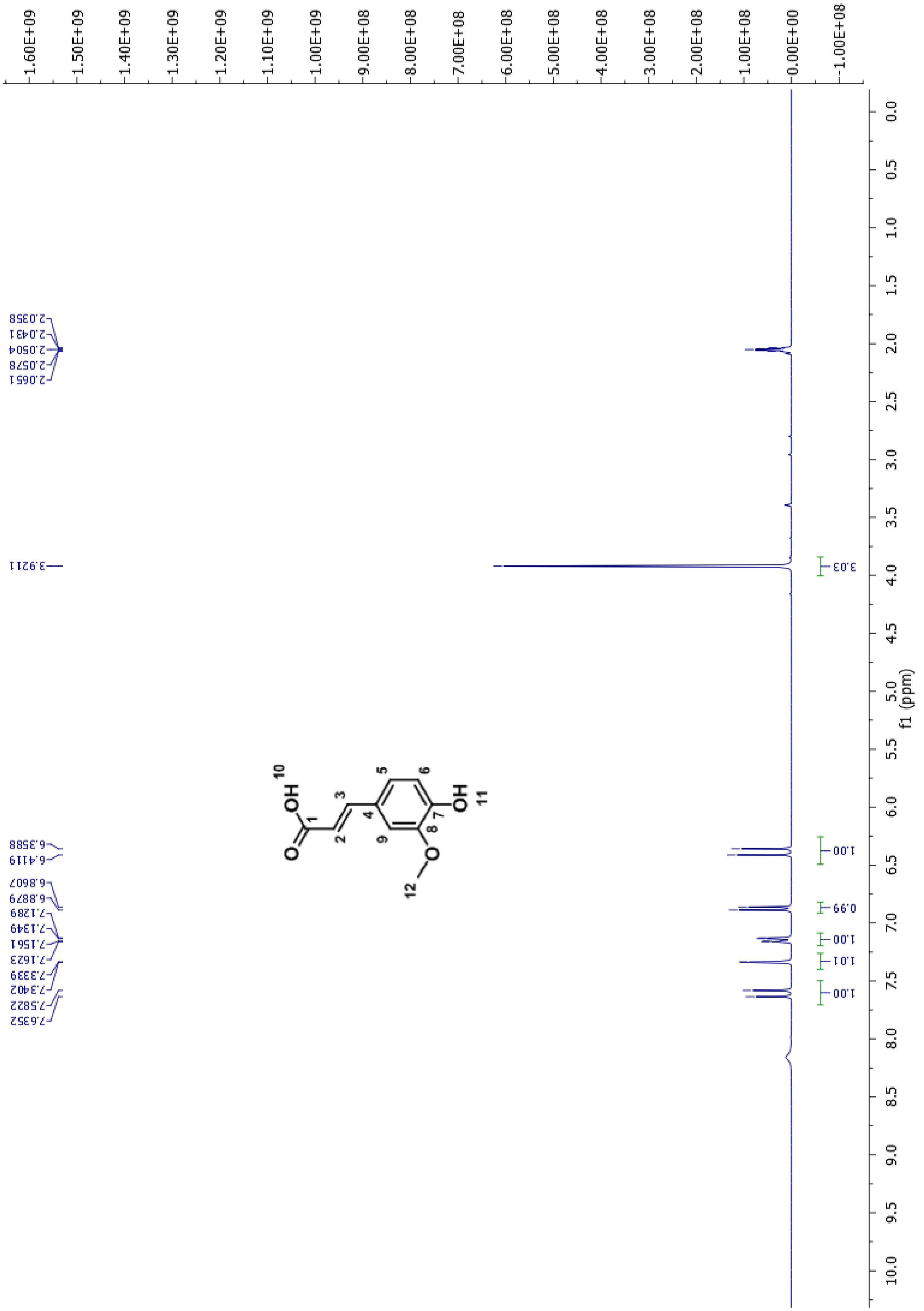

^{13}C NMR spectrum of Ferulic acid

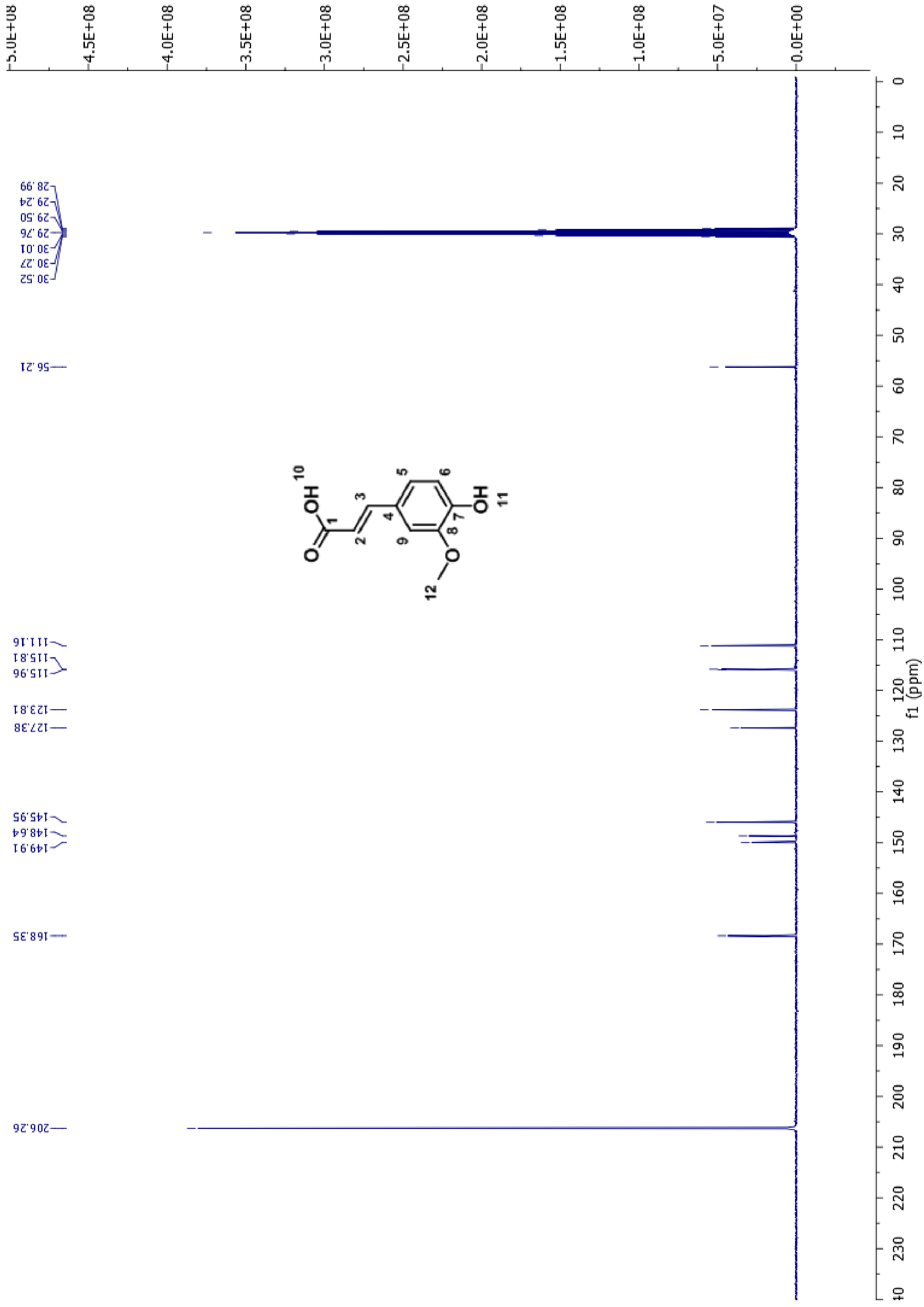

^{1}H NMR spectrum of the crude mixture of 5-hydroxyvanillin. 3.4-Dihydroxy-5-methoxycinnamic acid and 3-Methoxy-5-vinylbenzene-1.2-diol

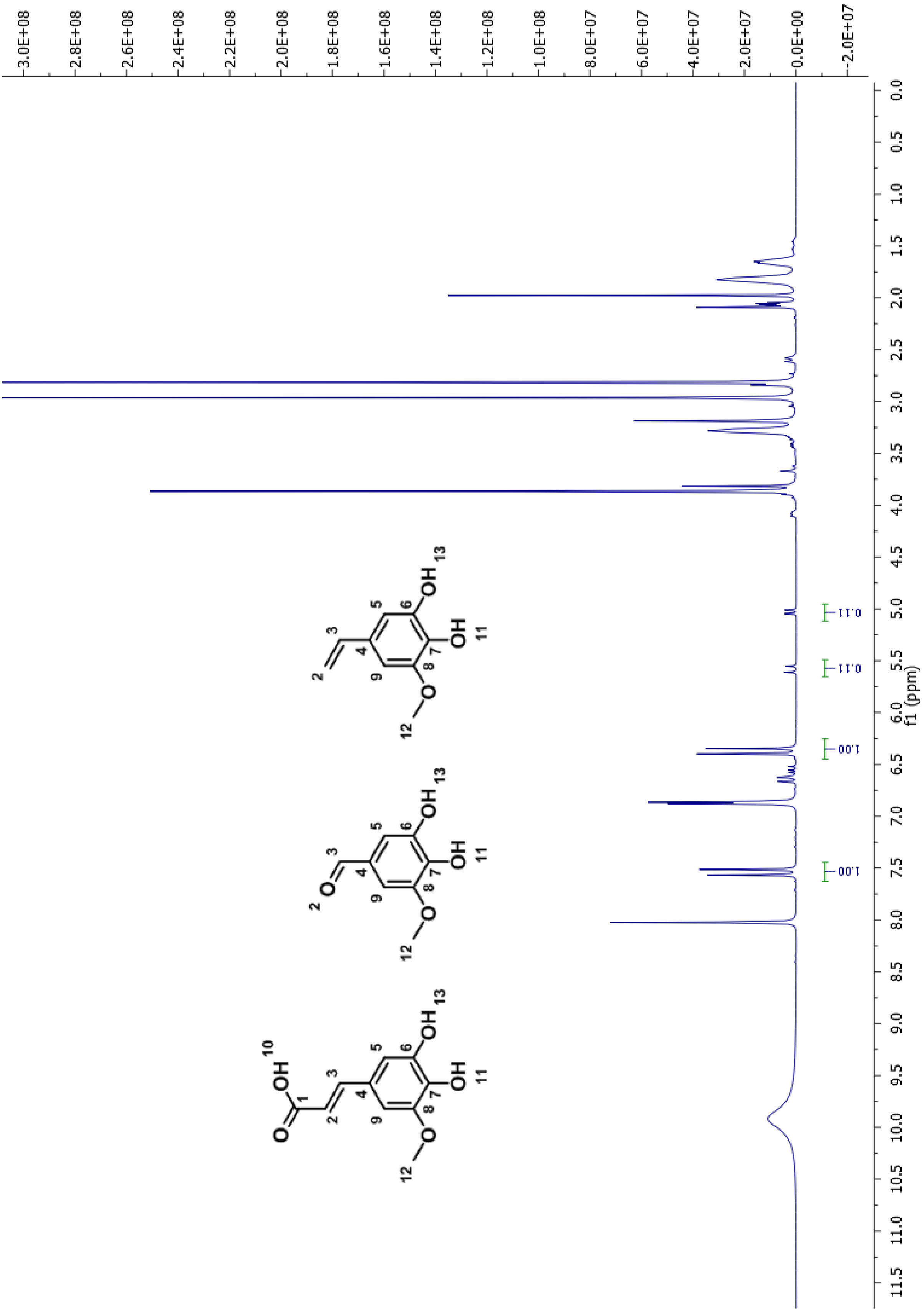

^{1}H NMR spectrum of 3.4-Dihydroxy-5-methoxycinnamic acid

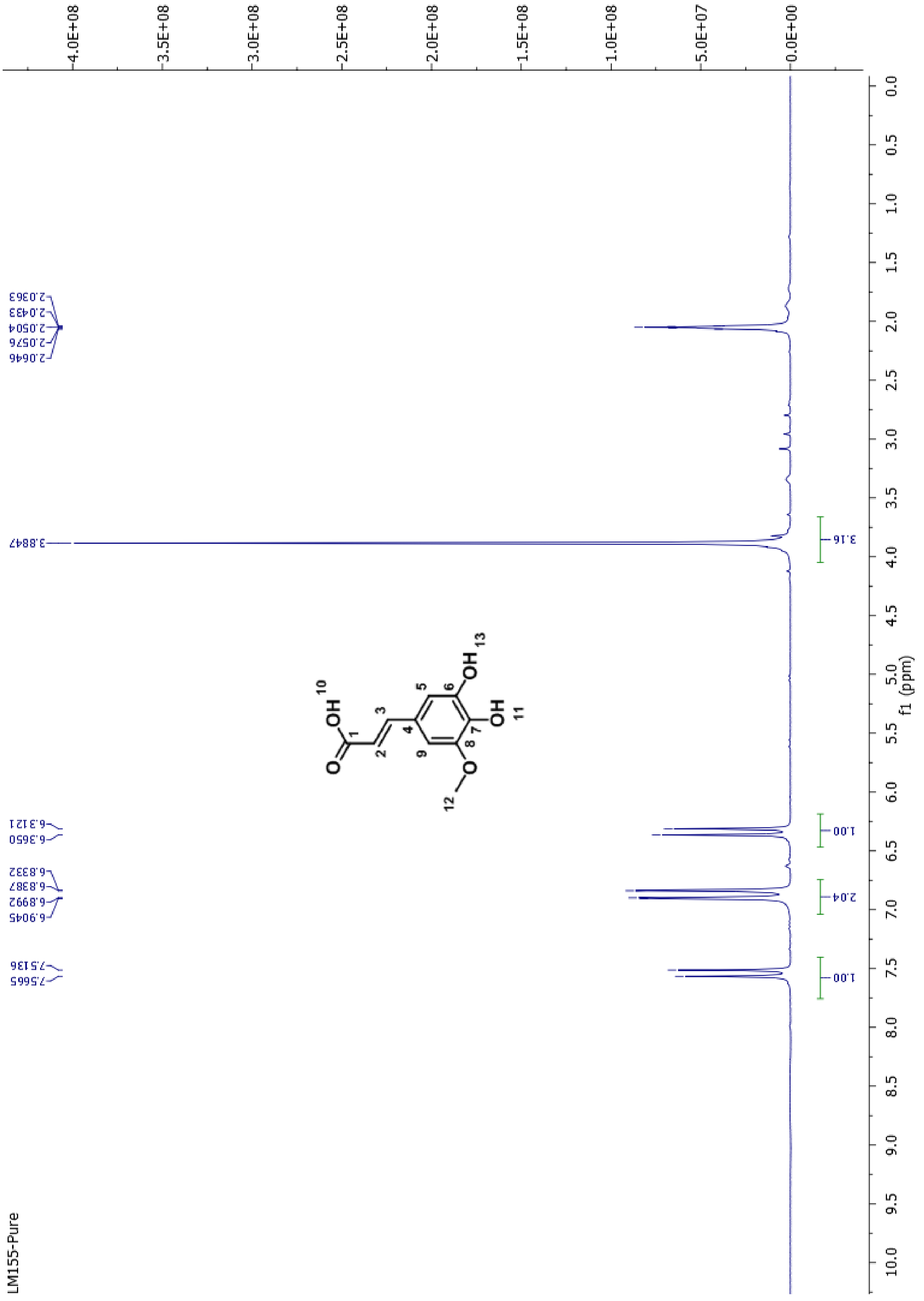

^{13}C NMR spectrum of 3.4-Dihydroxy-5-methoxycinnamic acid

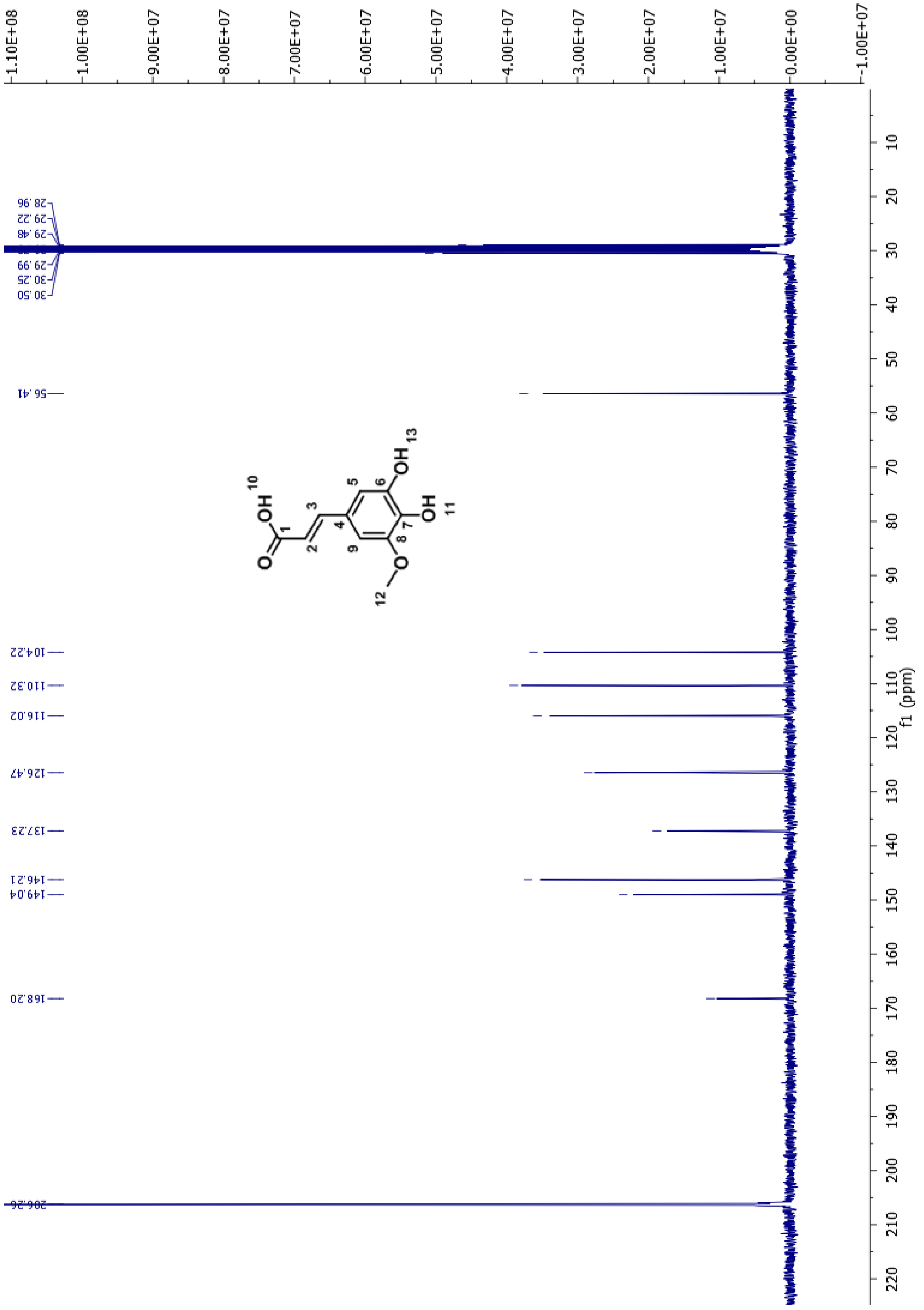

^{1}H NMR spectrum of the crude mixture of Syringaldehyde. Sinapic acid and 2.6-dimethoxy-4-vinylphenol

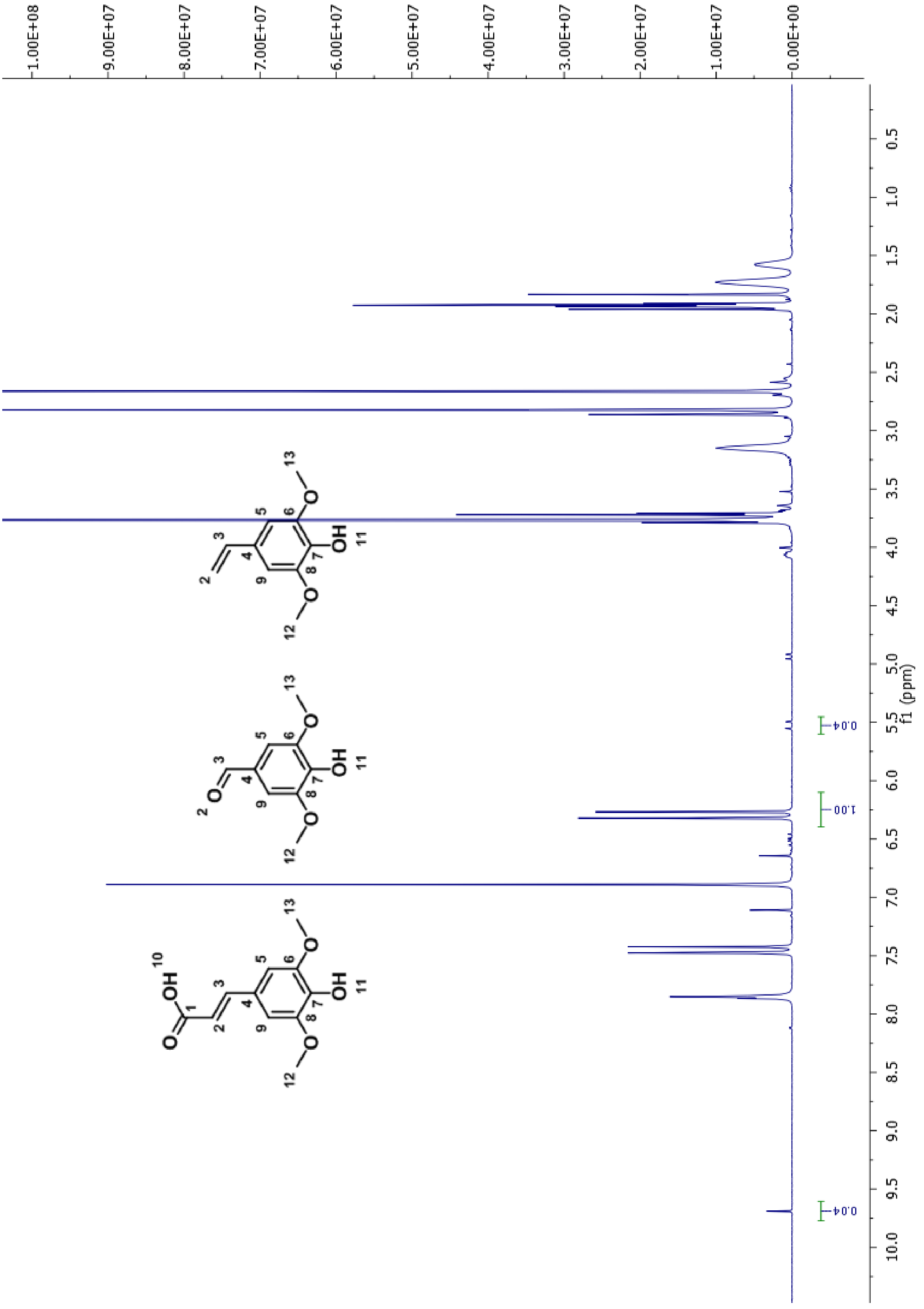

^{1}H NMR spectrum of Sinapic acid

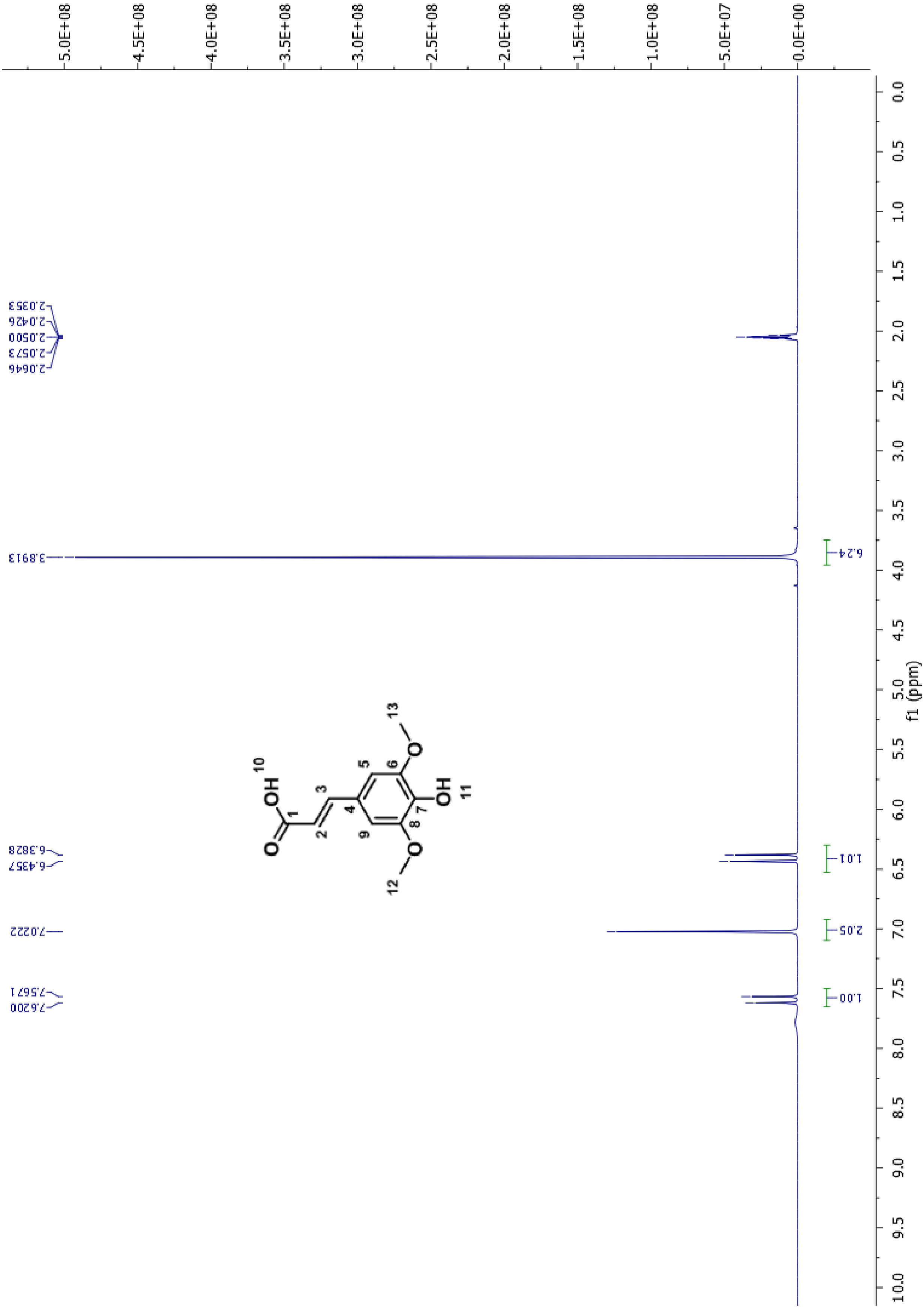

^{13}C NMR spectrum of Sinapic acid

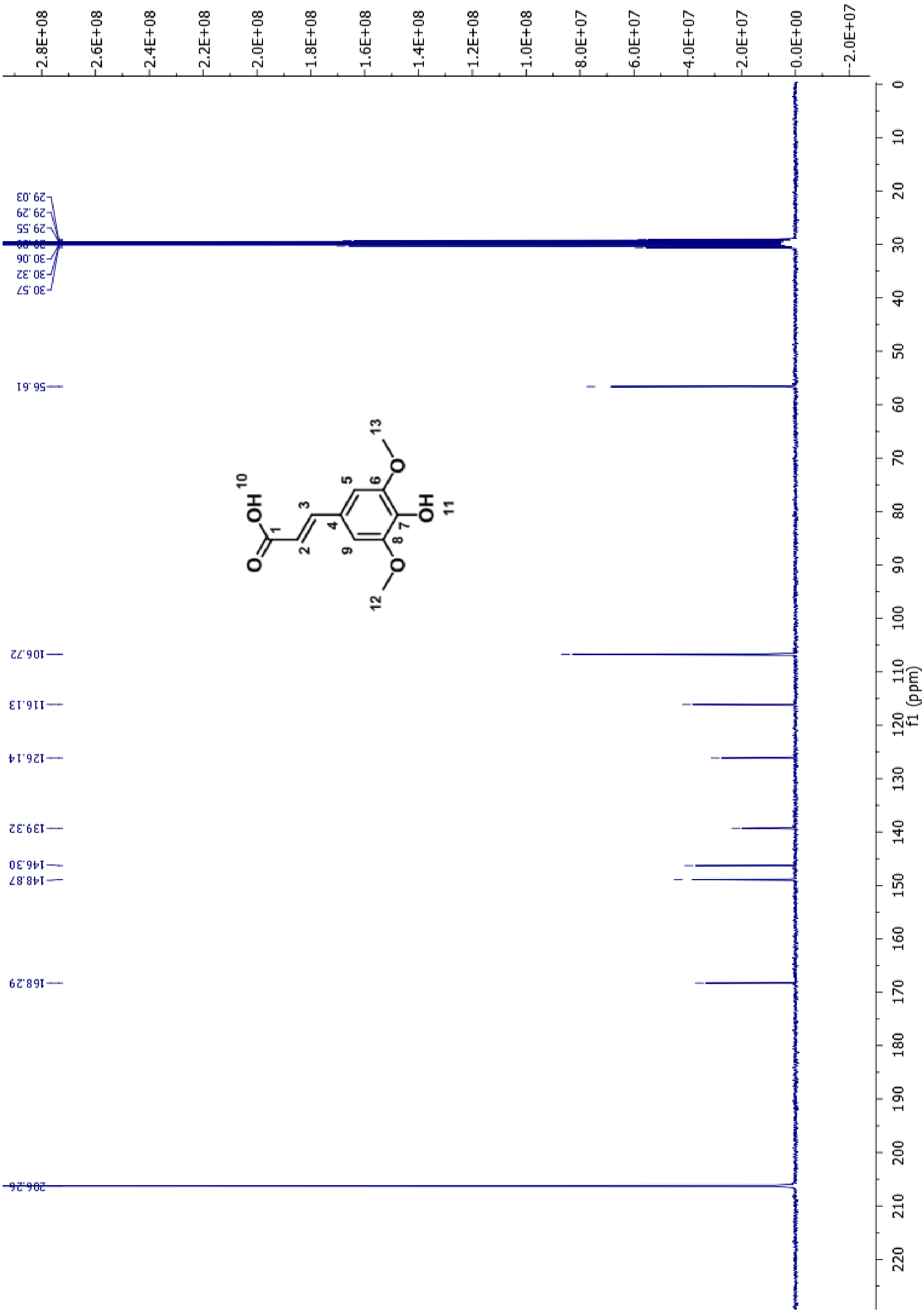

Supplement: Supplementary file 1 [file Data_Sheet_1.PDF]
